# Neuronal conversion of single-chain tissue-type plasminogen activator into its two-chain form: implications in neurodevelopment, learning, and memory

**DOI:** 10.1038/s41419-025-08132-8

**Published:** 2025-11-07

**Authors:** Hortense Triniac, Simon Lebatard, Valerie Roussel, Charlotte Lechevallier, Laurent Lebouvier, Denis Vivien, Benoit D. Roussel

**Affiliations:** 1 Université de Caen Normandie, Inserm, Normandie Université, Physiopathology and Imaging of Neurological Disorders (PhIND) UMR-S 1237, BB@C Institute, GIP Cyceron, Bvd Becquerel, BP 5229, 14074 Caen, France; 2 Department of Clinical Research, Caen-Normandie University Hospital, Caen, France

**Keywords:** Proteases, Cellular neuroscience

## Abstract

Tissue-type plasminogen activator (tPA) is a serine protease expressed in the central nervous system (CNS) that exhibits various effects, from neurodevelopment to learning and memory processes. tPA is secreted in its single-chain form (sc-tPA) and can be cleaved into a two-chain form (tc-tPA), with the two isoforms displaying sometimes opposite effects within the CNS. Using Alexa Fluor-conjugated recombinant tPA and complementary pharmacological approaches, we evaluated the ability of brain cells to process sc- into tc-tPA and the mechanisms involved. Our data revealed that neurons are the main brain cells capable to cleave sc-tPA into tc-tPA. This process occurs in three steps: 1) plasminogen binds to the cell surface of cortical neurons; 2) sc-tPA activates plasminogen into plasmin; 3) the generated plasmin cleaves sc-tPA into tc-tPA. The cleavage of tPA requires its Kringle 2 domain and is independent of plasminogen LBS. This cleavage mechanism represents a new modulation of tPA’s functions within the CNS.

## Introduction

Tissue-type plasminogen activator (tPA) is a glycoprotein that belongs to the serine protease family, mainly known for its role in fibrinolysis through its ability to activate plasminogen into active plasmin [1]. Unlike most serine proteases, tPA is synthesized as an active enzyme, rather than a zymogen, and is expressed by many different cells including endothelial cells [2], hepatocytes [3], glial cells [4–6], and neurons [6–8]. tPA consists of five domains, which from its *N*-terminal end to its *C*-terminal end are the finger domain, EGF-like domain, Kringle 1 domain, Kringle 2 domain, and serine protease domain. Released in a single-chain form (sc-tPA) into the circulation [9] or the extracellular space, it can be then cleaved within the Kringle 2 domain (hydrolysis of the Arg^275^-Ile^276^ peptide bond) into a two-chain form (tc-tPA) by plasmin or kallikreins [10]. Following this process, the heavy and light chains of tPA remain linked to each other via a disulfide bridge (Cys^299^-Cys^430^), conferring to sc- and tc-tPA a range of functions varying according to its conformation. In the vascular compartment, while tc-tPA is five time more proteolytically active than sc-tPA in the absence of fibrin, both forms exhibit the same catalytic activity when fibrin is present [11, 12].

tPA is also present in the brain parenchyma, where it interacts with various neuronal receptors and exerts diverse effects depending on its form. For instance, tPA plays a role in synaptic growth and neuronal migration during development [13–16]. Its effects are not solely mediated by extracellular matrix degradation, but also involve interactions with receptors such as the *N*-methyl-D-Aspartate receptor (NMDAR) [16]. Moreover, tPA is crucial for the late phase of long-term potentiation (l-LTP) in mice, as gene mutations disrupt l-LTP [17], and inhibition of the protease activity impairs l-LTP whereas direct application of tPA enhances it [18]. A possible mechanism underlying these effects is the interaction of tPA with the GluN1 subunit of NMDAR *via* its lysine binding site (LBS) located within its Kringle 2 domain [19–21]. Interestingly, the form of tPA determines its effects on NMDAR. In models of NMDA stimulations, only the interaction of sc-tPA with NMDAR promotes calcium influx, whereas tc-tPA does not [12]. In contrast, tc-tPA down-regulates signaling of NMDAR containing the GluN2B subunit through a crosstalk with c-MET receptor leading to a decrease of the NMDAR-mediated calcium influx, while sc-tPA does not [22].

As many roles of tPA depend on its form, it is of importance to understand the mechanisms of its cleavage in the brain parenchyma. In this study, we demonstrate a rapid and specific conversion of sc-tPA into tc-tPA occurring only at the cell surface of mature cortical neurons, among all the cell types tested. This enzymatic process is mediated by the plasmin generated from the activation of neuronal-bound plasminogen by tPA.

## Material and methods

### Chemicals

Foetal bovine serum (FBS; FB-1001), and horse serum (HS; HO-290) were purchased from Biosera (Nuaillé, France). Calcium chloride (CaCl_2_; C1016), HEPES 2X (51558), Dulbecco’s modified eagle’s medium (DMEM; D5671), poly-D-lysine (P6407), cytosine β-D-arabinoside (C1768), *ε*-Aminocaproic acid (EACA; A2504), *trans*-4-(Aminomethyl)cyclohexanecarboxylic acid (TXA; 857653), aprotinin from bovine lung (A62279), α2-antiplasmin (SRP6313), Hank’s balanced salt solution (HBSS; H-8264), dextran (31398), collagenase/dispase (10269638001), Tosyl Lysin Chloromethyl Ketone (TLCK; T-7254), amino acids BME (B-6766), basic fibroblast growth factor (bFGF; F-0291), vitamins (B-6891), collagen I (CLS354236-1EA), and Carboxypeptidase B (08039852001) were purchased from Sigma-Aldrich (Saint Louis, MO, USA). Laminin (23017015), molecular biology grade water (46000CI), AlexaFluor^488^ (A2000), Bovine Serum Albumin (BSA) conjugated to AlexaFluor^555^ (A34786), DMEM GlutaMAX (32430027), Neurobasal medium (21103049), B-27 supplement (17504044), sodium pyruvate (11360039), L-Glutamine (25030024), and DNase I (NC9082558) were purchased from Thermo Fisher Scientific (Waltham, MA, USA). Human plasmin (HPlasmin) and Glu-plasminogen (HPg 2001) were purchased from Enzyme Research Laboratories (South Bend, IN, USA). Anti-Plg-RKT monoclonal antibody [23] was generously provided by Dr. Lindsey Miles (The Scripps Research Institute, La Jolla, CA, USA).

### Production of recombinant ΔK2-tPA

The rat ΔK2-tPA vector was generated as previously described [24]. Briefly, the rat tPA c-DNA sequence was amplified by PCR with the following primers: 5′-CAGGCCGCACGTGGAGTCCTGAGTTGGTCCCTTAGG-3′ and 5′-TCCACCTGCGGCCTG-3′, with a His_6_ tag at the *N*-terminal position. PCR products were inserted into a pcDNA5/FTR vector and final constructs were automatically sequenced. HEK293T cells were transfected with calcium phosphate (250 mM CaCl_2_, HEPES 1X, molecular biology grade water). 18 hours after the transfection, the medium was replaced by DMEM supplemented with 1% sodium pyruvate, 0.5% L-Glutamine, 3 mg/L aprotinin. The supernatant containing the recombinant proteins was collected at 5 days and 8 days after the transfection and centrifugated at 4600 g during 20 minutes at 4°C. Purification of the mutant proteins was performed with a fast protein liquid chromatography using Nickel-Nitrilo-triAcetic affinity columns (Ni-NTA, GE Healthcare) and a mobile phase imidazole. Positives elutions were pooled and dialyzed during 48 hours in 0.3 M HEPES buffer, pH 7.4.

### Labelling of recombinant proteins with AlexaFluor

tPA (Actilyse®; Boehringer Ingelheim) was dialyzed in a 0.3 M HEPES buffer, pH 8.4, at 4°C for 48 h (the buffer was renewed every 12 h), in order to remove excipients, in particular arginine. Different batches containing initial proportions of sc-tPA varying from 80% to 60% were used. tPA and ΔK2-tPA were then mixed with 1 mg of AlexaFluor^488^ succinimidyl ester, previously suspended in 100 µL of dimethyl sulfoxide, overnight at 4°C with constant stirring. To remove excess AlexaFluor, the solution was dialyzed in a 0.3 M pH 7.4 HEPES buffer.

### Primary neurons culture with serum

Primary cultures of cortical neurons were prepared from fetal mice (embryonic day 14) as previously described [25]. Briefly, cortices were dissected and mechanically dissociated in DMEM before seeding at 1.10^6^ cells/mL on 24-well plates coated with poly-D-lysine (0.1 mg/mL) and laminin (0.02 mg/mL), in DMEM supplemented with 5% FBS, 5% HS, and 1 mM L-glutamine (Supplementary Table 1). Cultures were maintained at 37°C in a humidified 5% CO_2_ atmosphere. Cytosine β-D-arabinoside (10 µM) was added after 3 days in vitro (DIV) to inhibit glial proliferation. Treatments were realized on either immature (7 DIV) or mature (12-14 DIV) cortical neurons. For lysed neurons, cells were frozen at -80°C overnight then thawed before incubation with tPA^488^ and/or BSA^555^.

### Primary neurons culture without serum

Cells were prepared as described above, and then cultured in Neurobasal medium supplemented with 2% B27 Supplement (Supplementary Table 1). Cultures were maintained at 37°C in a humidified 5% CO_2_ atmosphere. Half the medium was changed every 3 days. Treatments were realized on mature (14 DIV) cortical neurons.

### Primary astrocyte culture

Primary cultures of cortical astrocytes were prepared from 1 to 3 days postnatal mice. Cerebral cortices were dissected and dissociated in DMEM. Cells were cultured in 24-well plate coated with poly-D-lysine (0.1 mg/mL), in DMEM supplemented with 10% FBS, 10% HS, and 1 mM L-glutamine (Supplementary Table 1). The medium was changed two times weekly. Experiments were performed when cells reached full confluency (7-9 DIV).

### Primary endothelial culture

Primary cultures of cerebral endothelial cells were prepared from adult mice (4 weeks old) as previously described [26] Briefly, meninge-free brains were collected and homogenized in buffer B (HBSS supplemented with 10 mM HEPES and 0.1% BSA) using a Dounce homogenizer (0.025 mm clearance, Wheaton). After addition of 30% dextran, tubes were vortexed, then centrifuged at 3000 g for 25 min at 4°C. The pellet was resuspended in buffer B and mechanically dissociated using a pipette. The obtained suspension was filtered through a 60 µm nylon mesh filter and digested with collagenase/dispase supplemented with DNase I and Tosyl-L-lysyl-chloromethane hydrochloride for 33 min at 37°C. The enzymatic reaction was stopped by adding cold buffer B, the solution was centrifuged at 1000 g for 7 min at RT and the cell pellet was resuspended in DMEM supplemented with 20% FBS, 2 mM L-Glutamine, 1% vitamins, 2% amino acids Basal Medium Eagle (BME), and 1 ng/mL Basic fibroblast growth factor (bFGF) (Supplementary Table 1), seeded in 6-well plates coated with 150 µg/cm^2^ collagen-I and incubated at 37°C in a humidified 5% CO_2_ atmosphere for 6 h. The medium was changed every 2 days. Experiments were performed when cells reached full confluency (7-9 DIV).

### PC-12 culture

PC-12 cells (rat adrenal gland cell line, CRL-1721, ATCC) were grown in RPMI-1640 (11875093, Gibco) supplemented with 12.5% HS and 2.5% FBS (Supplementary Table 1), maintained at 37 °C in a humidified 5% CO_2_ atmosphere. To induce PC-12 differentiation into a “neuron-like” phenotype, cells were seeded in DMEM F12 (11039021, Gibco) supplemented with 10% FBS, 2 mM HEPES, and 44 mM sodium bicarbonate for 2 hours. Medium was then replaced by DMEM F12 supplemented with 50 ng/mL nerve growth factor (NGF; N2513, Sigma) and 0.25% BSA (Supplementary Table 1) and renewed every two days until neurites growth after 7-10 days.

### HEK-293T culture

Human embryonic kidney (HEK)-293T (CRL-3216, ATCC) were grown in T75 in DMEM GlutaMAX supplemented with 10% FBS (Supplementary Table 1) and maintained at 37°C in a humidified 5% CO_2_ atmosphere. The medium was changed every two days. Experiments were performed when cells reached confluency (3-5 DIV).

### Conditioned medium

Mature cortical neurons were washed three times with 800 µL of serum-free DMEM and incubated at 37°C/5% CO_2_ for two hours. The supernatant was then collected and filtered before incubation of tPA^488^.

### Cell treatments

Before experiments, all cells were washed three times with 800 µL of serum-free DMEM and at 37°C/5% CO_2_ for two hours before adding treatments. Drugs were subsequently added to the serum-free media, and the corresponding vehicle (water or DMSO) was added to control conditions.

### Electrophoresis

Cell supernatants were concentrated at 4°C with 10 kDa MWCO Amicon centrifugal filter unit (UFC5010, Sigma-Aldrich), before migration on a 10% SDS-PAGE gel (4568033, Bio-Rad, Hercules, CA, USA), visualized on the ChemiDoc MP Imaging System (Bio-Rad), and analysed with Image Lab software. The sc-tPA band intensity was divided by the tc-tPA band intensity to obtain the sc-tPA/tc-tPA ratio. For each experiment, the ratio was normalized to the control condition (incubation on neurons in serum free DMEM at 37°C for less than a minute).

### Plasmin activity assay and laser scanning confocal microscopy

Plasmin activity was assessed by adding a fluorescent plasmin substrate at 10 µM (Sensolyte AFC Plasmin Assay Kit Fluorometric, AnaSpec, Fremont, CA, USA) on 12-14 DIV cortical neurons seeded on 8 well chamber slide (80826, Ibidi, Gräfelfing, Germany), in the presence or not of tPA^488^ and plasmin. This assay consists in the enzymatic cleavage of a fluorogenic peptide by plasmin. Once cleaved, the peptide releases the AFC (7-amido-4-trifluoromethylcoumarin) fluorophore whose fluorescent signal reflects plasmin activity. Laser-scanning confocal microscopy was performed on living cultures at 37°C, using a Leica TCS SP8 confocal microscope (Leica Microsystems SAS; Leica, Wetzlar, Germany) equipped with an Argon Gas laser and a X40 NA = 1.3 oil immersion objective. Cortical neurons were scanned with 380 and 488 nm laser lines to detect the plasmin substrate and tPA^488^, respectively. All images were acquired using the same parameters, including laser intensity and power, exposure time, and gain. High-resolution images (2048×2048, 12 bits) were analyzed with similar display adjustments using the ImageJ software.

### tPA-GGACK

H-Glu-Gly-Arg-chloromethyl Ketone (GGACK, 4018214, VWR, Radnor, USA) was incubated with tPA at a 4:1 ratio at room temperature for 5 h and dialyzed for 24 hours in 0.3 M HEPES pH 7.4 with Slide-A-Lyser MINI Dialysis 10 kDa MWCO device (88401, Thermo Fisher) to remove the unbound GGACK. The concentration was determined with a Implen™ NanoPhotometer™ N50 (Thermo Fisher). The complete inhibition of tPA was checked by an activity assay.

### Kinetics calculation

**Fig. 1:**
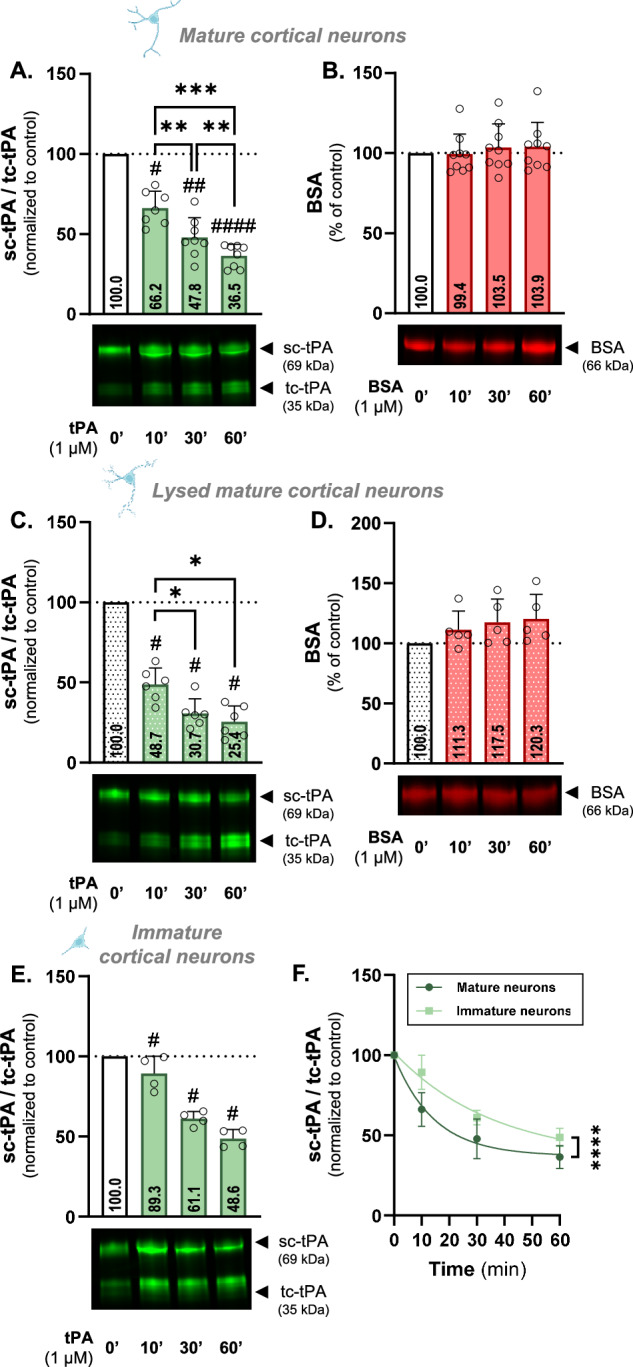
sc-tPA is cleaved into tc-tPA by primary cortical neurons via a mechanism acquired during cell maturation. **A** Densitometric quantification of the ratio sc-tPA^488^/tc-tPA^488^ (1 µM) normalized to stain free in % of control condition in supernatant of living mature (12 DIV) cortical neurons and representative electrophoresis. **B** Densitometric quantification of BSA^555^ (1 µM) normalized to stain free in % of control condition in supernatant of living mature cortical neurons representative electrophoresis. **C** Densitometric quantification of the ratio sc-tPA^488^/tc-tPA^488^ (1 µM) normalized to stain free in % of control condition in supernatant of lysed by freezing mature cortical neurons representative electrophoresis. **D** Densitometric quantification of BSA^555^ (1 µM) normalized to stain free in % of control condition in supernatant of lysed by freezing mature cortical neurons representative electrophoresis. **E** Densitometric quantification of the ratio sc-tPA^488^/tc-tPA^488^ (1 µM) normalized to stain free in % of control condition in supernatant of living immature (7 DIV) cortical neurons and representative electrophoresis. **F** Kinetic representation of the ratio sc-tPA^488^/tc-tPA^488^ in mature (12 DIV) and immature (7 DIV) cortical neurons. Data are represented as mean ± SD; *n* = 8 (A, B); *n* = 6 (C, D); * *p* < 0.05; ** *p* < 0.01; **** *p* < 0.0001; # *p* < 0.05 compared to control; ## *p* < 0.01 compared to control; #### *p* < 0.0001 compared to control; One-sample Wilcoxon test, Mann-Whitney test, F test.

**Fig. 2:**
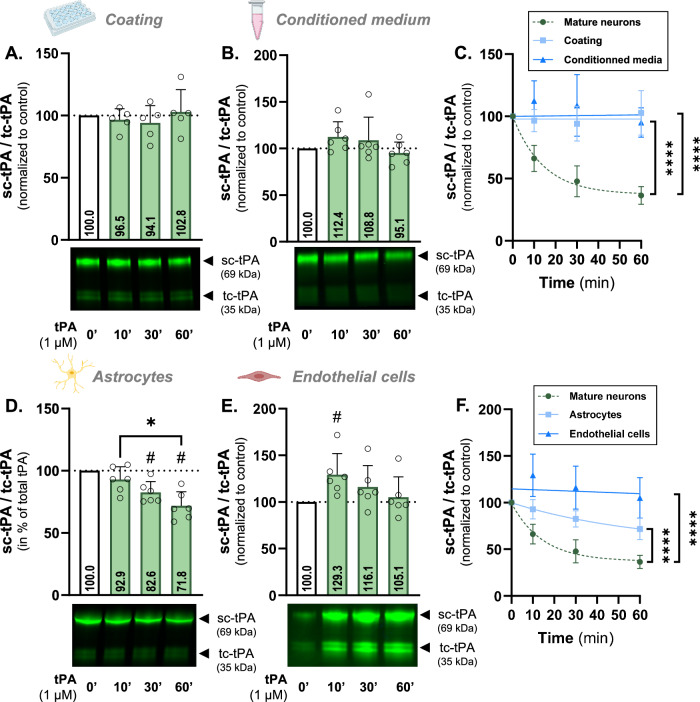
Cleavage of sc-tPA into tc-tPA is specific to cortical neurons and requires their maturation. **A** Densitometric quantification of the ratio sc-tPA^488^/tc-tPA^488^ (1 µM) normalized to stain free in % of control condition in supernatant of cell-free coated wells and representative electrophoresis. **B** Densitometric quantification of the ratio sc-tPA^488^/tc-tPA^488^ (1 µM) normalized to stain free in % of control in cell-free supernatant of mature cortical neurons and representative electrophoresis. **C** Kinetic representation of the ratio sc-tPA^488^/tc-tPA^488^ in cell-free coated wells (coating), and cell-free supernatant (conditioned medium) compared to the previously investigated mature neurons. **D** Densitometric quantification of the ratio sc-tPA^488^/tc-tPA^488^ (1 µM) normalized to stain free in % of control condition in supernatant of murine astrocytes and representative electrophoresis. **E** Densitometric quantification of the ratio sc-tPA^488^/tc-tPA^488^ (1 µM) normalized to stain free in % of control condition in supernatant of murine primary cerebral endothelial cells and representative electrophoresis. **F** Kinetic representation of the ratio sc-tPA^488^/tc-tPA^488^ in astrocytes and cerebral endothelial cells compared to the previously investigated mature neurons. Data are represented as mean ± SD; *n* = 5 (**A**, **C**); *n* = 6 (B-F); * *p* < 0.05; **** *p* < 0.0001; # *p* < 0.05 compared to control; One-sample Wilcoxon test, Mann-Whitney test, F test.

**Fig. 3:**
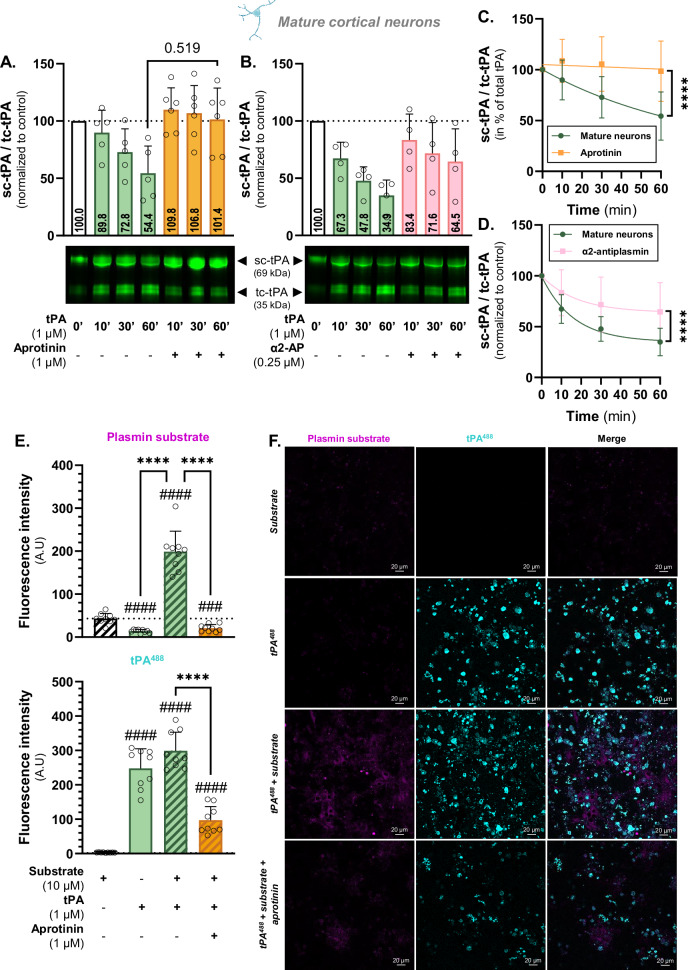
Plasmin enzymatic activity is responsible for neuronal cleavage of sc-tPA. **A** Densitometric quantification of the ratio sc-tPA^488^/tc-tPA^488^ (1 µM) in the presence or not of aprotinin (1 µM) normalized to stain free in supernatant of living mature cortical neurons and representative electrophoresis. **B** Densitometric quantification of the ratio sc-tPA^488^/tc-tPA^488^ (1 µM) in the presence or not of α2-antiplasmin (0.25 µM) normalized to stain free in supernatant of living mature cortical neurons and representative electrophoresis. **C** Kinetic representation of the ratio sc-tPA^488^/tc-tPA^488^ in mature neurons in the presence or not of aprotinin. **D** Kinetic representation of the ratio sc-tPA^488^/tc-tPA^488^ in mature neurons in the presence or not of α2-antiplasmin. **E** Quantitative analysis of fluorescence in mature cortical neurons exposed for 60 minutes to tPA^488^ (1 µM) and fluorescent plasmin substrate (10 µM) in the presence or not of aprotinin (1 µM). **F** Representative confocal microscopy images of mature cortical neurons exposed for 60 minutes to tPA^488^ (cyan) and fluorescent plasmin substrate (magenta) in the presence or not of aprotinin. Data are represented as mean ± SD; *n* = 5 (**A**, **C**, **D**); *n* = 4 (**B**, **C**); *n* = 9 (**E**, **F**); **** *p* < 0.0001; #### *p* < 0.0001 compared to control; One-sample Wilcoxon test, Mann-Whitney test, F test; scale bar = 20 µm.

**Fig. 4:**
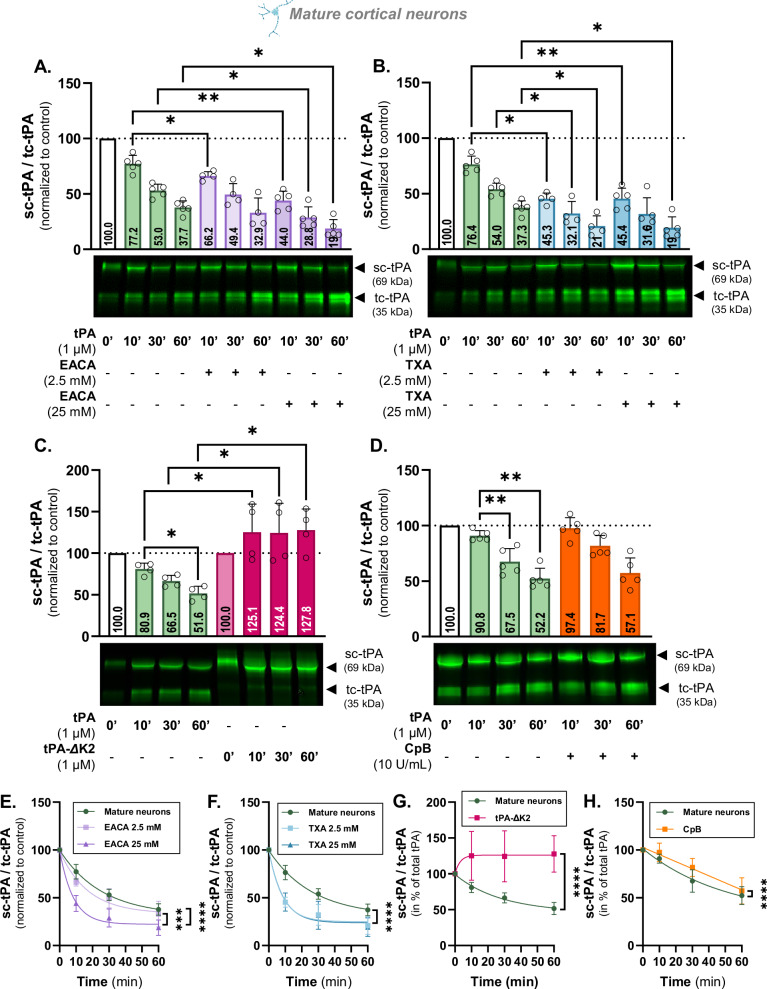
tPA cleavage requires tPA Kringle 2 domain and is independent from plasminogen LBS. **A** Densitometric quantification of the ratio sc-tPA^488^/tc-tPA^488^ (1 µM) in the presence or not of EACA (2.5 and 25 mM) normalized to stain free in supernatant of living mature cortical neurons and representative electrophoresis. **B** Densitometric quantification of the ratio sc-tPA^488^/tc-tPA^488^ (1 µM) in the presence or not of TXA (2.5 and 25 mM) normalized to stain free in supernatant of living mature cortical neurons and representative electrophoresis. **C** Densitometric quantification of the ratio sc-tPA^488^/tc-tPA^488^ (1 µM) and the Kringle 2-deleted sc-ΔK2-tPA^488^/ΔK2-tc-tPA^488^ normalized to stain free in supernatant of living mature cortical neurons and representative electrophoresis. **D** Densitometric quantification of the ratio sc-tPA^488^/tc-tPA^488^ (1 µM) in the presence or not of CpB (10 U/mL) normalized to stain free in supernatant of living mature cortical neurons and representative electrophoresis. **E** Kinetic representation of the ratio sc-tPA^488^/tc-tPA^488^ in mature neurons in the presence or not of EACA. **F** Kinetic representation of the ratio sc-tPA^488^/tc-tPA^488^ in mature neurons in the presence or not of TXA. **G** Kinetic representation of the ratio sc-tPA^488^/tc-tPA^488^ and sc-tPA-ΔK2^488^/tc-tPA-ΔK2^488^ in mature neurons. **H** Kinetic representation of the ratio sc-tPA^488^/tc-tPA^488^ in mature neurons, in the presence or not of CpB. Data are represented as mean ± SD; *n* = 5 (**A**, **B**, **D**–**F**, **H**); *n* = 4 (**A**, **B**, **C**, **E**–**G**); * *p* < 0.05; ** *p* < 0.01; *** *p* < 0.001; **** *p* < 0.0001; One-sample Wilcoxon test, Mann-Whitney test, F test.

**Fig. 5:**
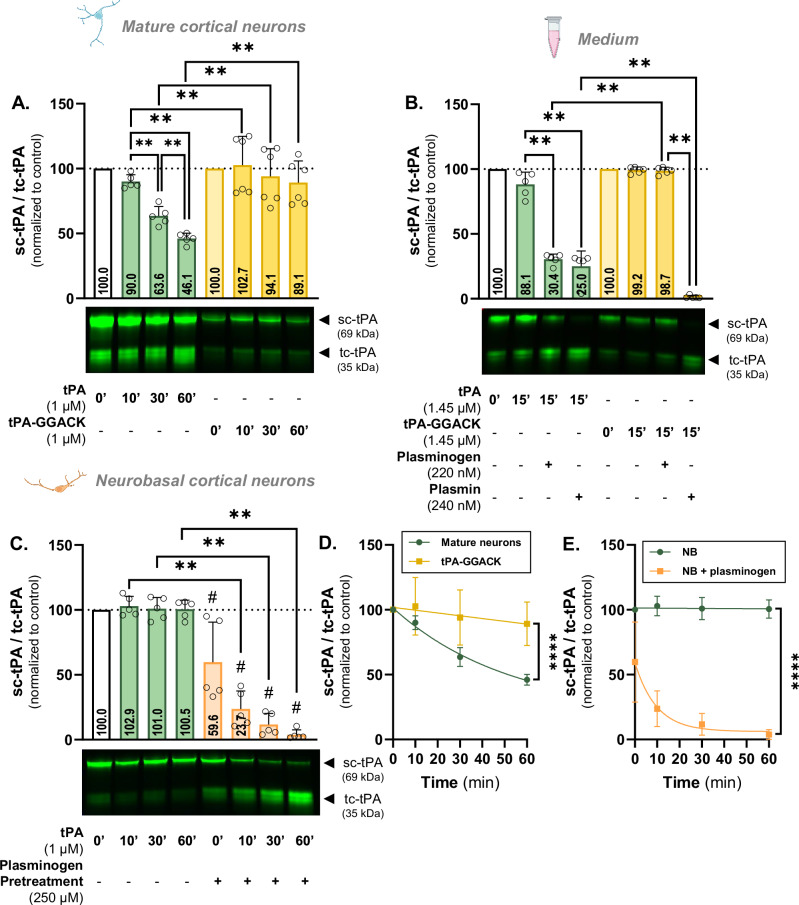
Neuronal cleavage involves tPA’s proteolytic activity. **A** Densitometric quantification of the ratio sc-tPA^488^/tc-tPA^488^ (1 µM) and the inactivated sc-tPA-GGACK^488^/tc-tPA-GGACK^488^ (1 µM) normalized to stain free in supernatant of living mature cortical neurons and representative electrophoresis. **B** Densitometric quantification of the ratio sc-tPA^488^/tc-tPA^488^ (1 µM) and the inactivated sc-tPA-GGACK^488^/tc-tPA-GGACK^488^ (1 µM) in the presence or not of plasminogen (250 nM) and plasmin (210 nM) normalized to stain free in DMEM and representative electrophoresis. **C** Densitometric quantification of the ratio sc-tPA^488^/tc-tPA^488^ (1 µM) with or without a 1 h pre-treatment with plasminogen (250 nM) normalized to stain free in supernatant of living mature cortical neurons cultured in neurobasal medium and representative electrophoresis. **D** Kinetic representation of the ratio sc-tPA^488^/tc-tPA^488^ and sc-tPA-GGACK^488^/tc-tPA-GGACK^488^ in mature neurons. **E** Kinetic representation of the ratio sc-tPA^488^/tc-tPA^488^ in mature neurons cultured in neurobasal medium with or without a plasminogen pretreatment. Data are represented as mean ± SD; *n* = 6 (**A**, **D**); *n* = 5 (**B**, **C**, **E**); ** *p* < 0.01; **** *p* < 0.0001; # *p* < 0.05 compared to control; One-sample Wilcoxon test, Mann-Whitney test, F test.

To calculate kinetics, ratio values obtained for each condition were plotted using MyCurveFit (https://mycurvefit.com) to generate kinetic curves. The initial linear portion of each curve (kinetic max) was used for statistical comparison in GraphPad Prism, using an F test on the slopes (simple linear regression; summary of comparisons provided in Supplementary Table 2). The kinetics in Fig. 1F, 2C, 2F, and Supplementary Fig. 1D were all compared to the same mature cortical neurons’ kinetic. Independent produced and purified tPA^488^ batches were used for Fig. 3C, D, 4E–H, and 5D, E, each condition including kinetics performed on mature cortical neurons serving as a reference. Notably, no significant differences in kinetics were observed between batches.

### Statistics

Statistical analyses were performed using GraphPad Prism software. Shapiro-Wilk tests were used to ensure a normal distribution. Comparisons of two data sets were performed using One sample Wilcoxon test for comparison with the control condition or Mann-Whitney test for comparison between experimental conditions. Comparisons for multiple data sets were performed using Kruskal-Wallis test with Dunn’s multiple comparison test.

## Results

### Cortical neurons acquire the ability to cleave sc-tPA into tc-tPA

The processing of tPA by cortical neurons and its underlying mechanisms were investigated by using human recombinant tPA labelled with AlexaFluor^488^ (tPA^488^). After incubating tPA^488^ with rinsed mature cortical neurons (12-14 DIV) for 10 minutes, the sc-tPA/tc-tPA ratio in the supernatant decreased from 100 in the control condition to 66.2 (*p* < 0.05; 33.8% decrease; Fig. 1A), reflecting enhanced tPA cleavage. Over time, this cleavage becomes more pronounced, with ratios decreasing to 47.8 and 36.5 at 30 and 60 minutes respectively (52.2% decrease *p* < 0.01 and 63.5% decrease *p* < 0.0001 compared to control; Fig. 1A). Bovine Serum Albumin (BSA), another circulating protein with a molecular weight comparable to tPA (66 kDa for BSA *vs*. 69 kDa for tPA), was used as a control. No cleavage or degradation was observed for BSA^555^ after incubation with mature cortical neurons (Fig. 1B), suggesting a specific mechanism for tPA.

To determine the role of cellular response in this process, we conducted the same experiments on mature neurons that had previously been lysed by a freeze/thaw cycle. tPA^488^ cleavage still happens, as indicated by reductions of 51.3% (48.7 vs. 100 in control; 0 < 0.05), 69.3% (30.7 vs. 100 in control; *p* < 0.05) and 74.6% (25.4 vs. 100 in control; *p* < 0.05) at 10, 30, and 60 minutes, respectively (Fig. 1C), suggesting a sustained cleavage over time. Following incubation with lysed mature cortical neurons, BSA^555^ remained uncleaved (Fig. 1D).

When incubated with rinsed immature cortical neurons (7 DIV) for 10, 30, and 60 minutes, we observed significant reductions in sc-tPA/tc-tPA ratio compared to the control condition: an 11.7% decrease after 10 minutes (from 100 to 89.3; *p* < 0.05), a 38.9% reduction at 30 minutes (from 100 to 61.1; *p* < 0.05), and a 51.4% decrease after 60 minutes (from 100 to 48.6; *p* < 0.05) (Fig. 1E). Even if a cleavage of sc- into tc-tPA is observed in immature neurons, its cleavage kinetics remains reduced compared to 12-14 DIV mature neurons (kinetics of cleavage, *p* < 0.0001; Fig. 1F).

These results demonstrate that mature cortical neurons rapidly cleave tPA, and this cleavage intensifies over time. The ability of lysed neurons to promote tPA’s cleavage also suggests that the conversion of sc- into tc-tPA occurs at the cell surface and does not require an intracellular process (endocytosis of tPA, or exocytosis of protease). The neuronal processing of tPA intensifies during maturation in vitro, then probably involving a membrane protein.

### tPA is only cleaved at the cell surface of cortical neurons

To check that the cleavage was not due to the serum-free media used for cell rinsing and tPA incubation, or by an activation surface provided by the coating (containing poly-D-lysine and laminin), we conducted experiments on conditioned media from mature neurons, or on media and coating without cells. In the presence of medium and coated wells, tPA^488^ was not cleaved (Fig. 2A), with cleavage kinetics that remains constant overtime and is significantly different from mature neurons previously investigated (*p* < 0.0001; Fig. 2C). This demonstrates that the medium is not able to cleave the tPA by itself, and that the coating does not provide an activation surface sufficient for triggering this mechanism.

We further explored whether elements present in mature neuron’s supernatant might play a role in this process. When incubated with conditioned medium, tPA^488^ was not cleaved (Fig. 2B) and displayed constant cleavage kinetics significantly different from neurons (*p* < 0.0001; Fig. 2C), reinforcing the need of a specific neuronal cell membrane as an activation surface.

This is also confirmed by other CNS cell types used to study tPA’s cleavage. Only a reduced cleavage of tPA^488^ was observed in primary cortical astrocytes (28.2% decrease after 60 minutes; *p* < 0.05; Fig. 2D and no cleavage in primary cerebral endothelial cells (Fig. 2E), with rates significantly lower than neurons for both cell types (*p* < 0.0001; Fig. 2F). Furthermore, tPA cleavage remained low in the presence of cells lines, such as the PC-12 rat cell line, either differentiated into a “neuron-like” phenotype (21.8% decrease after 60 minutes; Supplementary Fig. 1A) or not (23.4% decrease at 60 minutes; *p* < 0.05; Supplementary Fig. 1B), and the human cell line HEK-293T (24.5% decrease at 60 minutes; *p* < 0.05; Supplementary Fig. 1C), all three displaying a significantly reduced cleavage rate compared to neurons (*p* < 0.0001; Supplementary Fig. 1D). Altogether, these results suggest a mechanism with higher affinity for the cellular surface of mature cortical neurons.

### Neuronal cleavage of tPA is dependent of plasmin

Plasmin is known to cleave single-chain tPA into its two-chain form [11, 27]. To investigate its role in this process, we used aprotinin, an inhibitor of plasmin, trypsin, chymotrypsin, and kallikrein, with no effect on tPA [28]; and α2-antiplasmin a specific inhibitor of plasmin. We previously demonstrated that 1 µM of aprotinin was sufficient to reverse the proteolytic effect of tPA on autophagy in this in vitro neuronal model [29].

When neurons were exposed to aprotinin the tPA cleavage was completely abolished, as shown by the sc-tPA/tc-tPA ratio over time. The reduction in the tPA ratio observed in normal conditions (from 100 to 89.8, 72.8, and 54.4 corresponding to diminution by 10.2%, 27.2%, and 45.6% at 10, 30, and 60 minutes, respectively) was absent in the presence of 1 µM aprotinin (from 100 to 109.8, 106.8, and 101.4 at 10, 30 and 60 minutes, respectively; Fig. 3A) with an abolished cleavage rate (*p* < 0001; Fig. 3C).

It has been previously reported that 0.25 µM α2-antiplasmin effectively inhibits plasmin-induced dendritic growth in hippocampal neurons [30]. When treated with the specific plasmin inhibitor, α2-antiplasmin, the cleavage was not blocked but strongly decreased as shown with ratios shifted from 67.3, 47.8, and 34.9 (diminution of 32.7%, 52.2%, and 65.1% compared to control condition) at 10, 30, and 60 minutes, respectively, to 83.4, 71.6, and 64.5 (16.6%, 28.4%, and 35.5% decrease in comparison with control) (Fig. 3B). The cleavage rate is significantly decreased in the presence of α2-antiplasmin (*p* < 0.0001; Fig. 3D), reflecting a reduced conversion of sc-tPA into tc-tPA but not a blockage.

We next studied the activity of plasmin using a fluorescent substrate. After 60 minutes of treatment with tPA^488^, the basal plasmin activity is increased and is consistently strongly inhibited in the presence of aprotinin (*p* < 0.0001; Fig. 3E and F). Interestingly, when checking the level of fluorescent tPA bound on cells, we observed a strong decrease in the aprotinin condition (*p* < 0.0001; Fig. 3E and F).

These findings demonstrate a system involving the enzymatic cleavage of tPA by plasmin.

### tPA cleavage requires its lysine binding site

Plasmin formation is inhibited by lysine analogues, such as EACA and TXA, by competing the LBS mainly on plasminogen. The efficacy of 25 mM EACA in inhibiting plasminogen conversion into plasmin and reversing tPA-induced excitotoxicity in neurons has been previously reported [12]. We therefore tested both lysine analogs, EACA and TXA, at concentrations of 25 mM and 2.5 mM. Surprisingly, the use of these analogues enhanced in a time dependent manner the cleavage of tPA^488^. Co-treatment with 2.5 mM EACA reduced the sc-tPA/tc-tPA ratio at 10 minutes (decrease shifted from 22.8% to 33.8%; *p* < 0.05; Fig. 4A). Treatment with 25 mM of EACA further reduced the sc-tPA/tc-tPA ratio at 10 minutes (44.0 vs. 77.2; *p* < 0.01), 30 minutes (28.8 vs. 53.0; *p* < 0.05), and 60 minutes (19.0 vs 37.7; *p* < 0.05) (Fig. 4A) and strongly increased tPA cleavage rate (*p* < 0.0001; Fig. 4E). Similar results were observed with 2.5 mM and 25 mM of TXA, which decreased the sc-tPA/tc-tPA ratios at 10 minutes, 30 minutes, and 60 minutes (*p* < 0.05 and *p* < 0.01; Fig. 4B), and enhanced cleavage rate (*p* < 0.0001; Fig. 4F). Lysine analogs such as EACA and TXA are known to strongly compete the binding of plasminogen through its LBS [31] but are poor competitor for tPA’s LBS [32]. The functional LBS of tPA is located on its Kringle 2 domain and is involved in the activation of plasminogen into active plasmin [33]. To explore the role of Kringle 2 in this cleavage mechanism, we incubated a fluorescent mutated tPA missing its Kringle 2 (tPA-ΔK2^488^) on mature cortical neurons. This tPA-ΔK2^488^, which is still cleavable by plasmin (Supplementary Fig. 4), remains uncleaved in the presence of neurons, being at 96% under its single-chain form regardless of the incubation time (*p* < 0.05; Fig. 4C) with an abolished cleavage rate (*p* < 0.0001; Fig. 4G). The Kringle 2 domain containing the functional LBS of tPA is thus mandatory for its cleavage, by activating the plasminogen into plasmin to promote the conversion of sc-tPA into tc-tPA.

For cellular plasminogen conversion into plasmin, plasminogen receptors are required [34, 35]. In each cell type, a specific subset of plasminogen binding sites, including a group of receptors exposing carboxy-terminal lysines on the cell surface (e.g. the receptor Plg-RKT), promotes plasminogen activation. These proteins are sensitive to carboxypeptidase B (CpB), an enzyme able to cleave carboxy-terminal lysines [34]. It has been previously demonstrated that 10 U/mL of CpB is sufficient to remove plasminogen bound to neurons in vitro [36]. When neurons are pretreated with CpB (10 U/mL), the cleavage of sc-tPA into tc-tPA is not modified (97.4 vs. 90.8 at 10 minutes; 81.7 vs. 67.5 at 30 minutes; and 57.1 vs. 52.2 at 60 minutes, non-significantly different; Fig. 4D). However, when looking at the kinetics of conversion, the cleavage rate is slightly inhibited (*p* < 0.0001, Fig. 4H), suggesting that the binding of plasminogen to neurons mainly relies on a CpB-insensitive plasminogen receptor. This is reinforced by the fact that the receptor Plg-RKT does not take part in this process. Indeed, no effect on tPA cleavage was observed in the presence of an antibody blocking the interaction between plasminogen and its receptor Plg-RKT (Supplementary Table 3).

Taken together, these data demonstrate that the cleavage of sc-tPA into tc-tPA involves the Kringle 2 domain of tPA and the binding of plasminogen independently from its LBS, via a receptor or a plasminogen binding protein not synthetized with a carboxy-terminal lysine.

### The proteolytic activity of tPA is required for its cleavage

GGACK is a modified small peptide that binds covalently to tPA in its active site, resulting in a complete irreversible inhibition of both sc- and tc-tPA [37, 38]. The proteolytically inactive tPA (tPA-GGACK) was not cleaved by cortical neurons with a significantly higher ratio compared to the active tPA at 10 minutes (102.7 vs. 90.0; *p* < 0.01), 30 minutes (94.1 vs. 63.6; *p* < 0.01), and 60 minutes (98.1 vs. 46.1; *p* < 0.01) (Fig. 5A) and a reduced cleavage rate (*p* < 0.001; Fig. 5D).

When incubated with plasminogen, tPA-GGACK remains single-chain (98.7 vs. 30.4; *p* < 0.01) and only plasmin induces its cleavage (0.8 corresponding to a 99.2% decrease compared to control, *p* < 0.01) (Fig. 5B).

Neurons cultured in a plasminogen-free medium (neurobasal medium, NB) were unable to cleave exogenous tPA, as indicated by the sc-tPA/tc-tPA ratio remaining unchanged (102.9, 101.0, and 100.5 at 10, 30, and 60 minutes, respectively). This ability is restored when cells are temporarily treated with plasminogen followed by rinsing and tPA incubation, resulting in ratios reduced by 76.3%, 92.7%, and 98.0% compared to the control condition at 10, 30 and 60 minutes, respectively (*p* < 0.05; Fig. 5C) and increase cleavage rate (*p* < 0.0001; Fig. 5E). These findings indicate that the binding of plasminogen, along with its conversion into plasmin by tPA, is essential for the cleavage of tPA by the generated plasmin.

We believe that the difference observed between neurons at DIV7 and DIV12–14 is due to the maturation and/or expression of a membrane receptor capable of binding plasminogen. However, as we have not yet identified this receptor, we cannot exclude the possibility that the increased neuritic arborization during development may enhance plasminogen binding, and thereby contribute to the observed differences in sc-tPA cleavage.

Taken together, these results expose a new property of cortical neurons that can enhance tPA proteolytic activity by promoting its cleavage through plasminogen activation.

## Discussion

The cleavage of sc-tPA at the cellular surface of cortical neurons occurs in three steps: 1) plasminogen binds to the neuronal surface; 2) tPA’s intrinsic activity activates plasminogen into plasmin; 3) the generated plasmin cleaves sc-tPA into tc-tPA.

Unlike most serine proteases, tPA is directly synthesized in an active form rather than as a zymogen, requiring additional regulatory mechanisms to control its activity. In the vascular compartment, circulating tPA is inactivated by binding to inhibitors, such as plasminogen activator inhibitor-1 (PAI-1), which can interact with both tPA isoforms [39]. In the CNS, tPA activity is further regulated by its cleavage, with sc- and tc-tPA exerting opposing effects of on neuronal NMDAR signaling [12, 22].

Lysine analogs are known to inhibit plasmin formation by competing with the LBS-dependent interaction between plasminogen and fibrin [40]. EACA, by blocking the high-affinity LBS on plasminogen and plasmin, has been shown to inhibit pro-urokinase- mediated plasminogen activation [31]. However, despite neuronal tPA cleavage being a plasmin-induced process, it was unexpectedly enhanced by both EACA and TXA. One possible explanation is that EACA stabilizes plasmin activity by preventing its degradation [41]. Additionally, EACA promotes the transition of plasminogen into an open conformation by binding to its low-affinity LBS [42], thereby exposing its cleavage site to tPA. Another possibility is that lysine analogs compete with plasminogen’s LBS involved in its binding to cellular receptors, leading to plasminogen release and increased spatial proximity to tPA. However, cleaving the carboxy-terminal lysines exposed on the cell surface does not really affect tPA cleavage, suggesting that binding of plasminogen to a receptor through its LBS is not the major process involved in tPA cleavage. The plasminogen-receptor group exposing carboxy-terminal lysines on the cell surface is sensitive to CpB and can be divided into two subgroups (1) proteins synthesized with *C*-terminal basic residues (i.e. lysine) that have well-established intracellular functions, such as α-enolase [43, 44], cytokeratin 8 [45, 46], S100A10 (in complex with annexin A2 within the annexin A2 heterotetramer) [47–49], TIP49a [50], and histone H2B [51]; and (2) proteins requiring proteolytic processing to expose a *C*-terminal basic residue (i.e. lysine), such as actin and others [52, 53]. However, another group of plasminogen-binding proteins, which are CpB-insensitive, are expressed at the eukaryotic cellular membrane and includes tissue factor [54], nonprotein gangliosides [55], αIIbβ3 [56, 57], αMβ2 [58, 59], and α5β1 [58], as well as amphoterin [60, 61] and GP330 [62, 63]. Our proteomic studies revealed that both CpB sensitive and insensitive binding proteins such as cytokeratin 8, annexin A2, histone H2B, and actin bind to tPA after neuronal incubation (Supplementary Table 4). However, the expression of the CpB-sensitive receptor, Plg-RKT, was detected in both cortical neurons and non-cleaving cells (here astrocytes; Supplementary Table 5), and co-treatment with an anti-plg-RKT antibody did not affect tPA cleavage (Supplementary Table 3). Taken together, these data suggest a supply of plasminogen that is mainly bound to the neuronal surface independently of its LBS.

The implication of other actors known to interact with tPA in the CNS such as GRP78 [64], LRP-1 receptor [65], MET receptor [22], and NMDA receptors [21, 66] has been explored via pharmacological modulation but none of them impacted the neuronal cleavage of tPA (Supplementary Table 3).

It is well-established that sc- and tc-tPA exert opposing effect on neuronal NMDAR signaling. While sc-tPA potentiates NMDAR signaling, tc-tPA does not [12, 24]. Moreover, tc-tPA has been shown to induce the internalization of NMDARs containing the GluN2B subunit, leading to decreased receptor signaling, an effect not observed with sc-tPA [22]. These studies were conducted using mature cortical neurons cultured under conditions similar to those of the present study. Under these conditions, tPA cleavage is expected to occur, potentially diminishing the differences between sc- and tc-tPA after 1 h of treatment. Notably, a non-cleavable form of tPA, generated via a point mutation at its cleavage site (R276S), did not enhanced NMDAR signaling [24]. This underscores the crucial role of the Kringle 2 domain in tPA-mediated modulation of NMDARs and explains the lack of effect of tc-tPA when its cleavage site remains intact. If neuronal tPA cleavage occurs more slowly than its immediate effects on NMDAR, this could account for the contrasting impacts of sc- and tc-tPA on receptor signaling. As previously mentioned, tPA plays a crucial role in development, as well as in learning and memory [13, 14, 16]. Plasminogen is highly expressed during early development, after which its expression declines [67]. Both plasminogen and tPA are known to contribute to cell migration and neuronal growth [67, 68]. The ratio of sc-tPA to tc-tPA may vary across different stages of brain development, but this has not yet been investigated. The regulation and mechanism of tPA conversion in neurons could therefore represent an additional step in brain maturation during development.

In conclusion, we report here a neuronal cleavage of tPA, specific to cortical neurons, which may serve as a modulatory mechanism for tPA activity within the CNS. Considering tPA’s different forms in future studies could provide deeper insights into its diverse functions not only in the parenchyma but also in the vascular compartment.

## Supplementary information

Supplementary Material and Methods

Supplementary Tables and Figures

Supplemenraty legends

## Data Availability

The datasets generated during and/or analyzed during the current study are available from the corresponding author on reasonable request.
