# Supplementary material for: Neuronal conversion of single-chain tissue-type plasminogen activator into its two-chain form: implications in neurodevelopment, learning, and memory
Source: Cell Death Dis. 2025 Nov 7;16(1):811. doi: 10.1038/s41419-025-08132-8 (PMC12594754; doi:10.1038/s41419-025-08132-8)
Supplement: Supplementary file 1 — Supplementary Material and Methods [file 41419_2025_8132_MOESM1_ESM.docx]

**SUPPLEMENTARY MATERIAL AND METHODS**

***Clot lysis assay***

Platelet poor plasma (PPP) from 6 healthy human donors (Établissement Français du Sang) were pooled and kept at -80°C. PPPs were thawed rapidly at 37°C prior clotting initiation with calcium chloride (25 mM final concentration), in the presence of rtPA (Alteplase; 0.25 mM) and/or CpB (1, 10, or 50 U/mL). Controls were done in all plates: a positive control (PPP + calcium chloride) and a negative control (PPP alone). Absorbance (405 nm) was measured every 30 seconds during 18 hours at 37°C with a plate reader (Spark, Tecan).

***Cross-linking and pull-down of tPA on neuronal plasma membrane***

Recombinant tPA (Alteplase) was biotinylated using NHS-PEG4-Biotin (21455, Thermo Fisher) according to the manufacturer’s protocol. After incubation on mature cortical neurons for 60 minutes at 37 °C, biotinylated tPA was cross-linked to the neuronal surface with BS(PEG)9 (21582, Thermo Fisher). Plasma membrane proteins were extracted using the Mem-PER Plus Membrane Protein Extraction kit (89842, Thermo Fisher) and proteins bound to biotinylated tPA were pulled-down with Dynabeads MyOne Streptavidin C1 beads (65001, Invitrogen).

***Plasma membrane and cytosolic proteins extraction in cleavable and non cleavable brain cells.***

Cytosolic and plasma membrane proteins were isolated from primary astrocytes, primary mature (12 DIV) and immature (7 DIV) cortical neurons using the Mem-PER Plus Membrane Protein Extraction kit (89842, Thermo Fisher).

***Proteomic analysis***

Mass spectrometry proteomic experiments were performed and analyzed in collaboration with the Proteogen platform (US EMerode, Université de Caen Normandie).
