# Supplementary material for: Neuronal conversion of single-chain tissue-type plasminogen activator into its two-chain form: implications in neurodevelopment, learning, and memory
Source: Cell Death Dis. 2025 Nov 7;16(1):811. doi: 10.1038/s41419-025-08132-8 (PMC12594754; doi:10.1038/s41419-025-08132-8)
Supplement: Supplementary file 2 — Supplementary Tables and Figures [file 41419_2025_8132_MOESM2_ESM.pptx]

## Slide 1
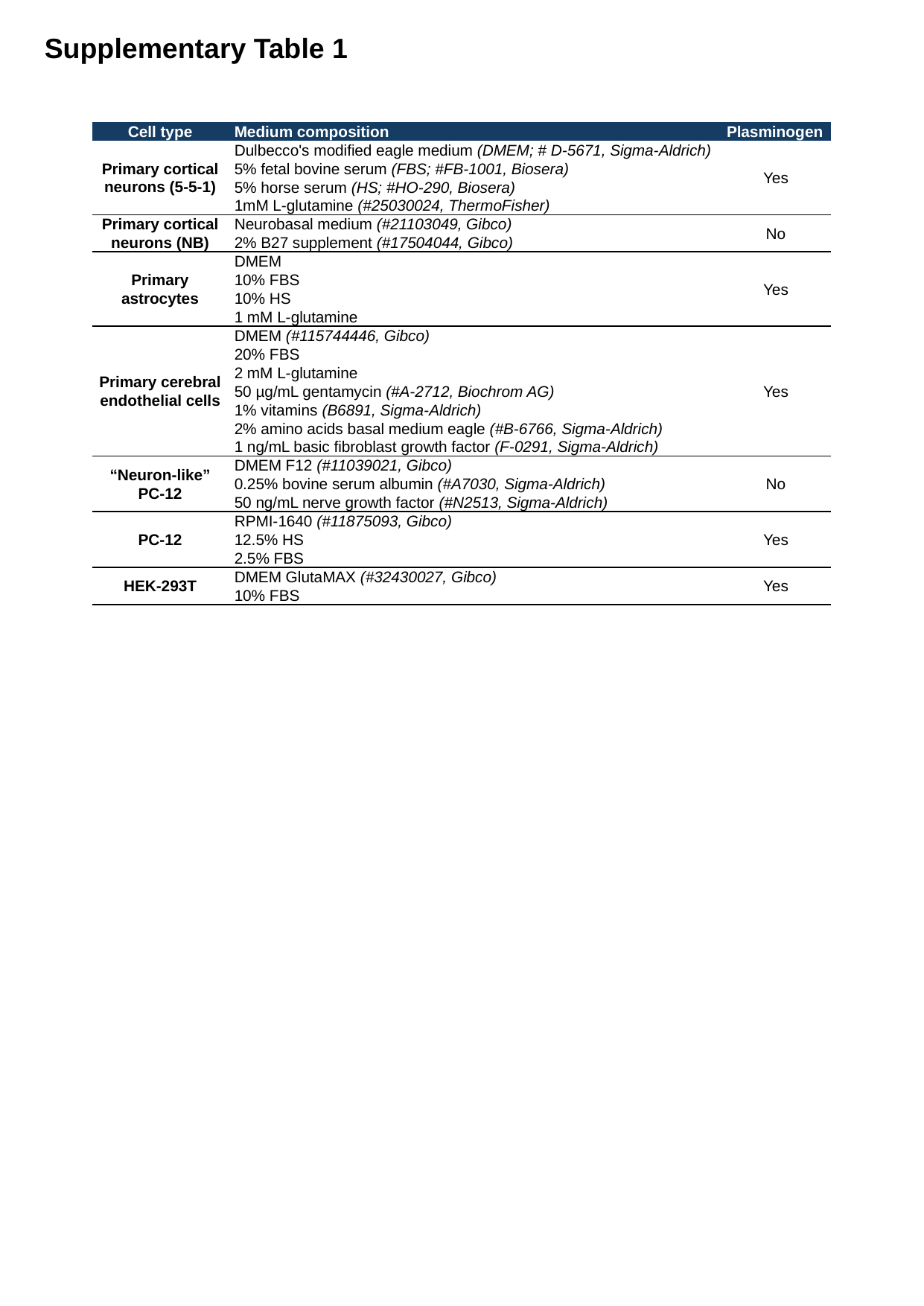

Supplementary Table 1
| Cell type | Medium composition | Plasminogen |
| --- | --- | --- |
| Primary cortical neurons (5-5-1) | Dulbecco's modified eagle medium (DMEM; # D-5671, Sigma-Aldrich) | Yes |
| | 5% fetal bovine serum (FBS; #FB-1001, Biosera) | |
| | 5% horse serum (HS; #HO-290, Biosera) | |
| | 1mM L-glutamine (#25030024, ThermoFisher) | |
| Primary cortical neurons (NB) | Neurobasal medium (#21103049, Gibco) | No |
| | 2% B27 supplement (#17504044, Gibco) | |
| Primary astrocytes | DMEM | Yes |
| | 10% FBS | |
| | 10% HS | |
| | 1 mM L-glutamine | |
| Primary cerebral endothelial cells | DMEM (#115744446, Gibco) | Yes |
| | 20% FBS | |
| | 2 mM L-glutamine | |
| | 50 µg/mL gentamycin (#A-2712, Biochrom AG) | |
| | 1% vitamins (B6891, Sigma-Aldrich) | |
| | 2% amino acids basal medium eagle (#B-6766, Sigma-Aldrich) | |
| | 1 ng/mL basic fibroblast growth factor (F-0291, Sigma-Aldrich) | |
| “Neuron-like” PC-12 | DMEM F12 (#11039021, Gibco) | No |
| | 0.25% bovine serum albumin (#A7030, Sigma-Aldrich) | |
| | 50 ng/mL nerve growth factor (#N2513, Sigma-Aldrich) | |
| PC-12 | RPMI-1640 (#11875093, Gibco) | Yes |
| | 12.5% HS | |
| | 2.5% FBS | |
| HEK-293T | DMEM GlutaMAX (#32430027, Gibco) | Yes |
| | 10% FBS | |

## Slide 2
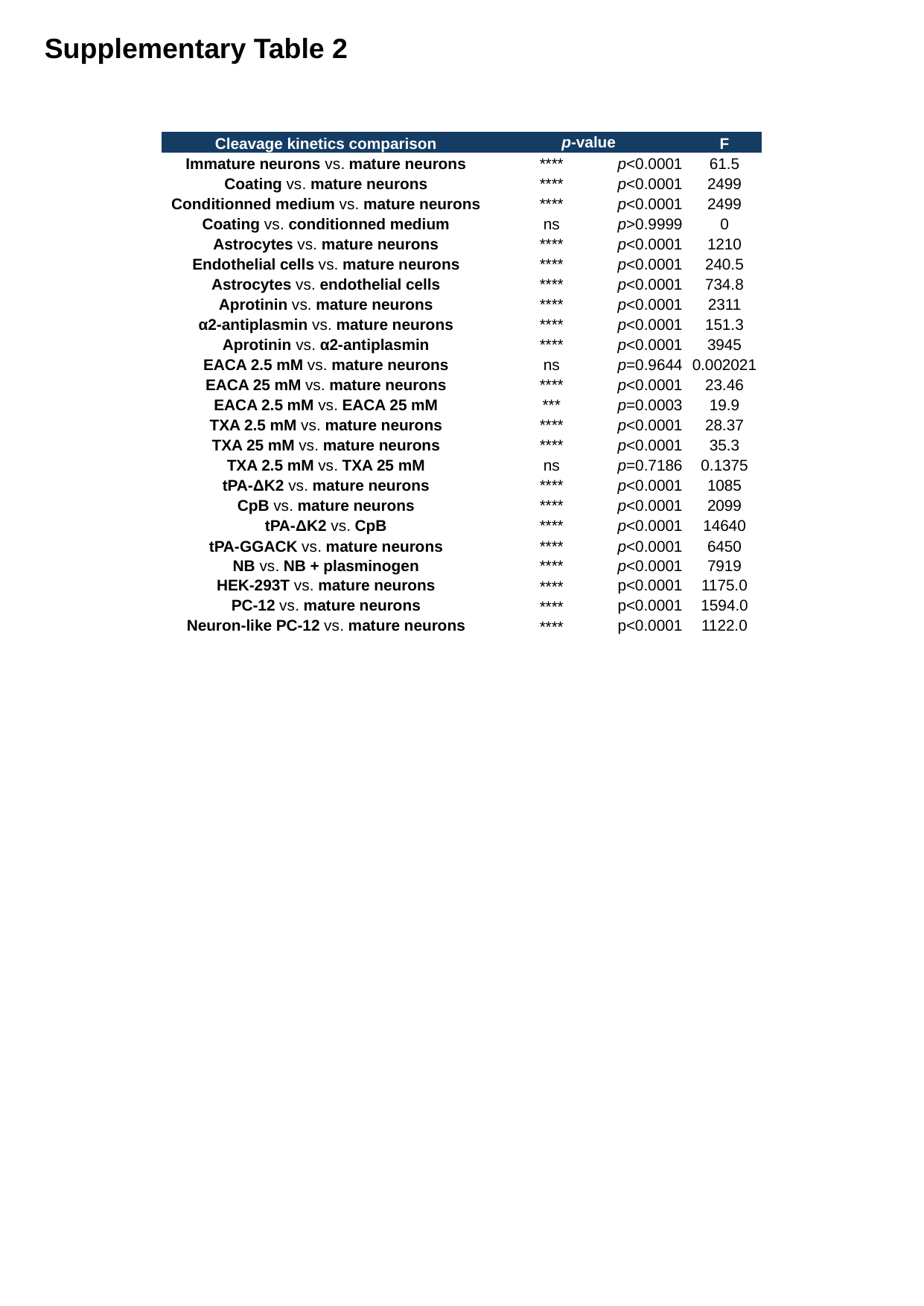

Supplementary Table 2
| Cleavage kinetics comparison | p-value | | F |
| --- | --- | --- | --- |
| Immature neurons vs. mature neurons | \*\*\*\* | p<0.0001 | 61.5 |
| Coating vs. mature neurons | \*\*\*\* | p<0.0001 | 2499 |
| Conditionned medium vs. mature neurons | \*\*\*\* | p<0.0001 | 2499 |
| Coating vs. conditionned medium | ns | p>0.9999 | 0 |
| Astrocytes vs. mature neurons | \*\*\*\* | p<0.0001 | 1210 |
| Endothelial cells vs. mature neurons | \*\*\*\* | p<0.0001 | 240.5 |
| Astrocytes vs. endothelial cells | \*\*\*\* | p<0.0001 | 734.8 |
| Aprotinin vs. mature neurons | \*\*\*\* | p<0.0001 | 2311 |
| α2-antiplasmin vs. mature neurons | \*\*\*\* | p<0.0001 | 151.3 |
| Aprotinin vs. α2-antiplasmin | \*\*\*\* | p<0.0001 | 3945 |
| EACA 2.5 mM vs. mature neurons | ns | p=0.9644 | 0.002021 |
| EACA 25 mM vs. mature neurons | \*\*\*\* | p<0.0001 | 23.46 |
| EACA 2.5 mM vs. EACA 25 mM | \*\*\* | p=0.0003 | 19.9 |
| TXA 2.5 mM vs. mature neurons | \*\*\*\* | p<0.0001 | 28.37 |
| TXA 25 mM vs. mature neurons | \*\*\*\* | p<0.0001 | 35.3 |
| TXA 2.5 mM vs. TXA 25 mM | ns | p=0.7186 | 0.1375 |
| tPA-ΔK2 vs. mature neurons | \*\*\*\* | p<0.0001 | 1085 |
| CpB vs. mature neurons | \*\*\*\* | p<0.0001 | 2099 |
| tPA-ΔK2 vs. CpB | \*\*\*\* | p<0.0001 | 14640 |
| tPA-GGACK vs. mature neurons | \*\*\*\* | p<0.0001 | 6450 |
| NB vs. NB + plasminogen | \*\*\*\* | p<0.0001 | 7919 |
| HEK-293T vs. mature neurons | \*\*\*\* | p<0.0001 | 1175.0 |
| PC-12 vs. mature neurons | \*\*\*\* | p<0.0001 | 1594.0 |
| Neuron-like PC-12 vs. mature neurons | \*\*\*\* | p<0.0001 | 1122.0 |

## Slide 3
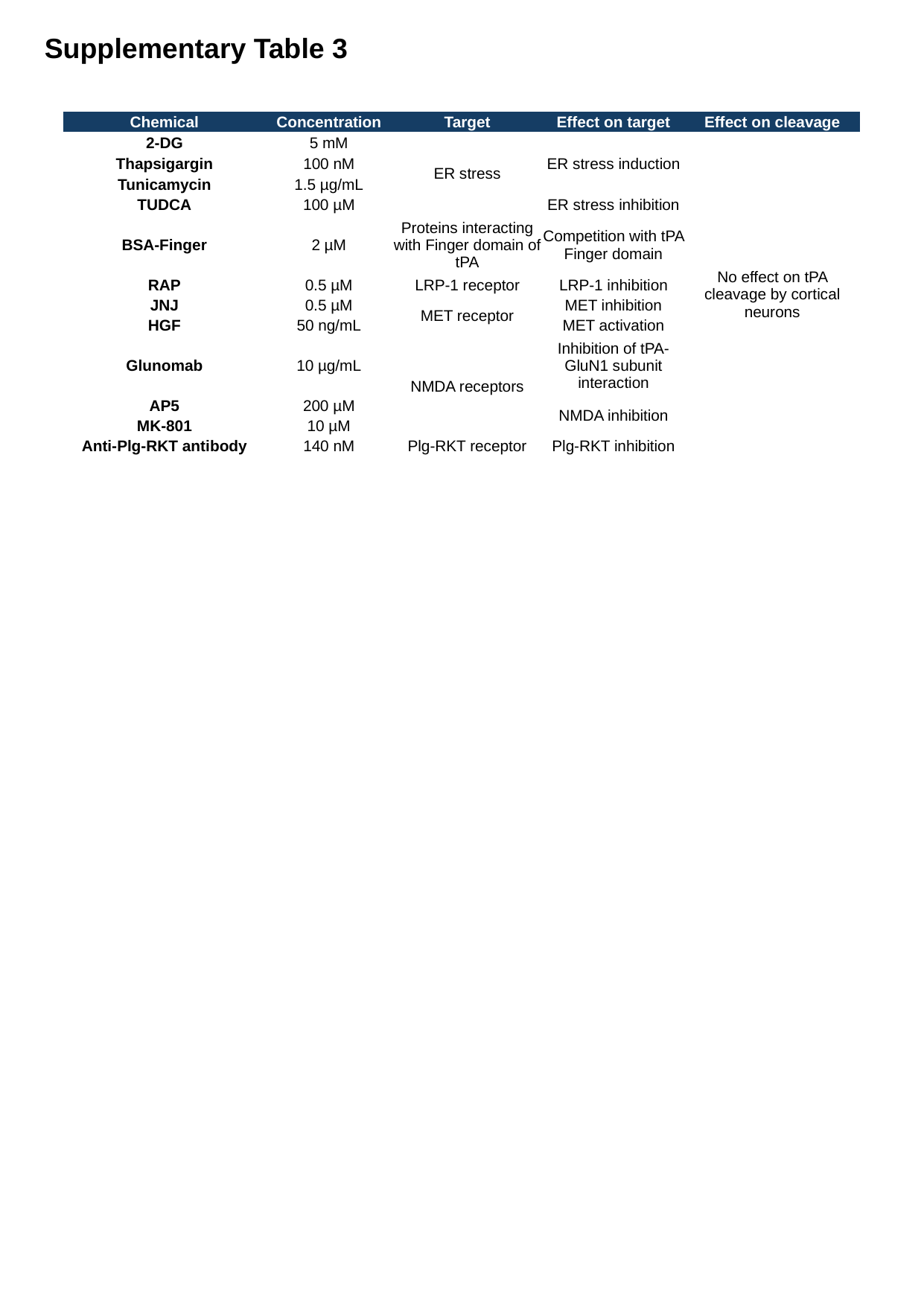

Supplementary Table 3
| Chemical | Concentration | Target | Effect on target | Effect on cleavage |
| --- | --- | --- | --- | --- |
| 2-DG | 5 mM | ER stress | ER stress induction | No effect on tPA cleavage by cortical neurons |
| Thapsigargin | 100 nM | | | |
| Tunicamycin | 1.5 µg/mL | | | |
| TUDCA | 100 µM | | ER stress inhibition | |
| BSA-Finger | 2 µM | Proteins interacting with Finger domain of tPA | Competition with tPA Finger domain | |
| RAP | 0.5 µM | LRP-1 receptor | LRP-1 inhibition | |
| JNJ | 0.5 µM | MET receptor | MET inhibition | |
| HGF | 50 ng/mL | | MET activation | |
| Glunomab | 10 µg/mL | NMDA receptors | Inhibition of tPA-GluN1 subunit interaction | |
| AP5 | 200 µM | | NMDA inhibition | |
| MK-801 | 10 µM | | | |
| Anti-Plg-RKT antibody | 140 nM | Plg-RKT receptor | Plg-RKT inhibition | |

## Slide 4
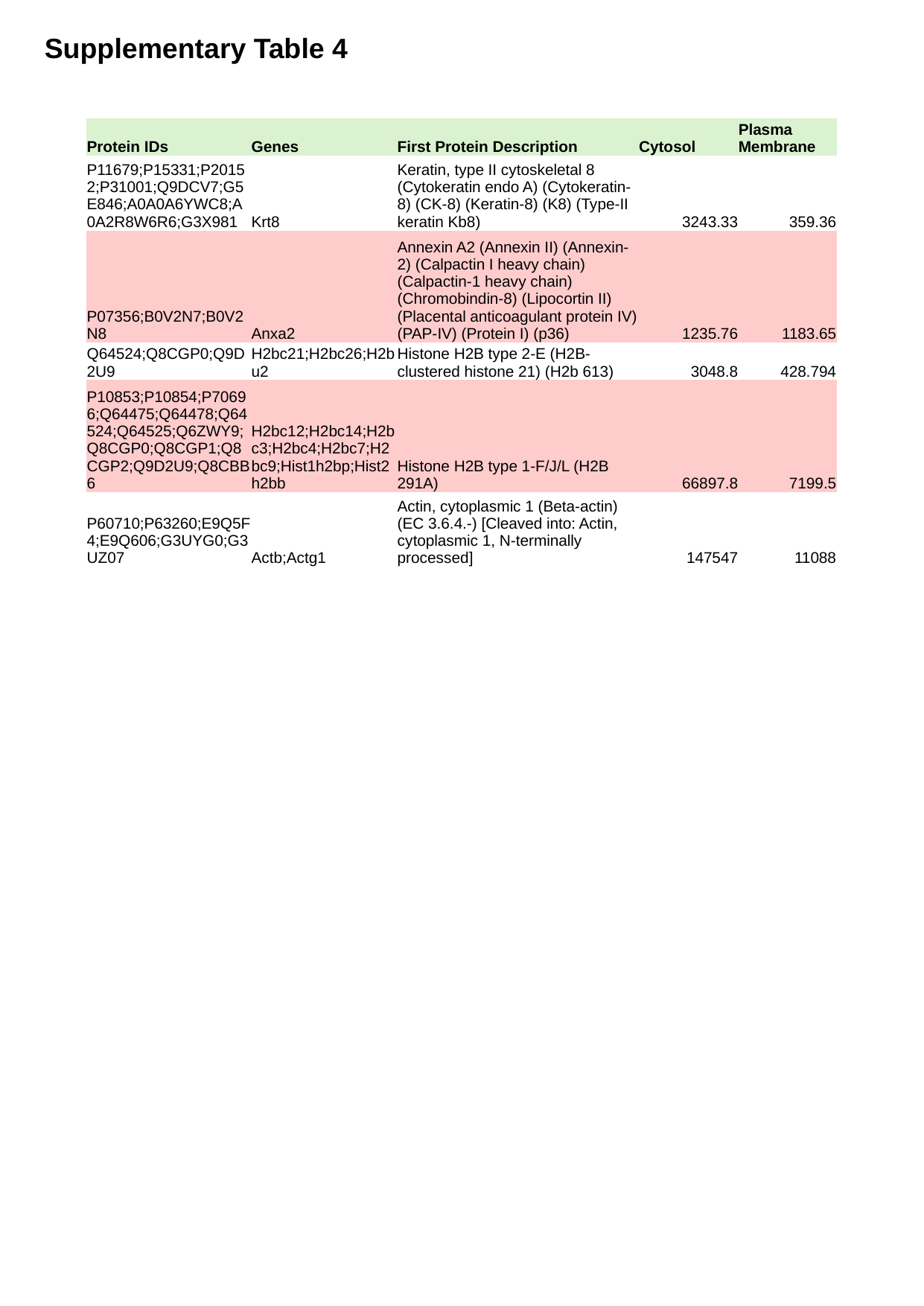

Supplementary Table 4
| Protein IDs | Genes | First Protein Description | Cytosol | Plasma Membrane |
| --- | --- | --- | --- | --- |
| P11679;P15331;P20152;P31001;Q9DCV7;G5E846;A0A0A6YWC8;A0A2R8W6R6;G3X981 | Krt8 | Keratin, type II cytoskeletal 8 (Cytokeratin endo A) (Cytokeratin-8) (CK-8) (Keratin-8) (K8) (Type-II keratin Kb8) | 3243.33 | 359.36 |
| P07356;B0V2N7;B0V2N8 | Anxa2 | Annexin A2 (Annexin II) (Annexin-2) (Calpactin I heavy chain) (Calpactin-1 heavy chain) (Chromobindin-8) (Lipocortin II) (Placental anticoagulant protein IV) (PAP-IV) (Protein I) (p36) | 1235.76 | 1183.65 |
| Q64524;Q8CGP0;Q9D2U9 | H2bc21;H2bc26;H2bu2 | Histone H2B type 2-E (H2B-clustered histone 21) (H2b 613) | 3048.8 | 428.794 |
| P10853;P10854;P70696;Q64475;Q64478;Q64524;Q64525;Q6ZWY9;Q8CGP0;Q8CGP1;Q8CGP2;Q9D2U9;Q8CBB6 | H2bc12;H2bc14;H2bc3;H2bc4;H2bc7;H2bc9;Hist1h2bp;Hist2h2bb | Histone H2B type 1-F/J/L (H2B 291A) | 66897.8 | 7199.5 |
| P60710;P63260;E9Q5F4;E9Q606;G3UYG0;G3UZ07 | Actb;Actg1 | Actin, cytoplasmic 1 (Beta-actin) (EC 3.6.4.-) [Cleaved into: Actin, cytoplasmic 1, N-terminally processed] | 147547 | 11088 |

## Slide 5
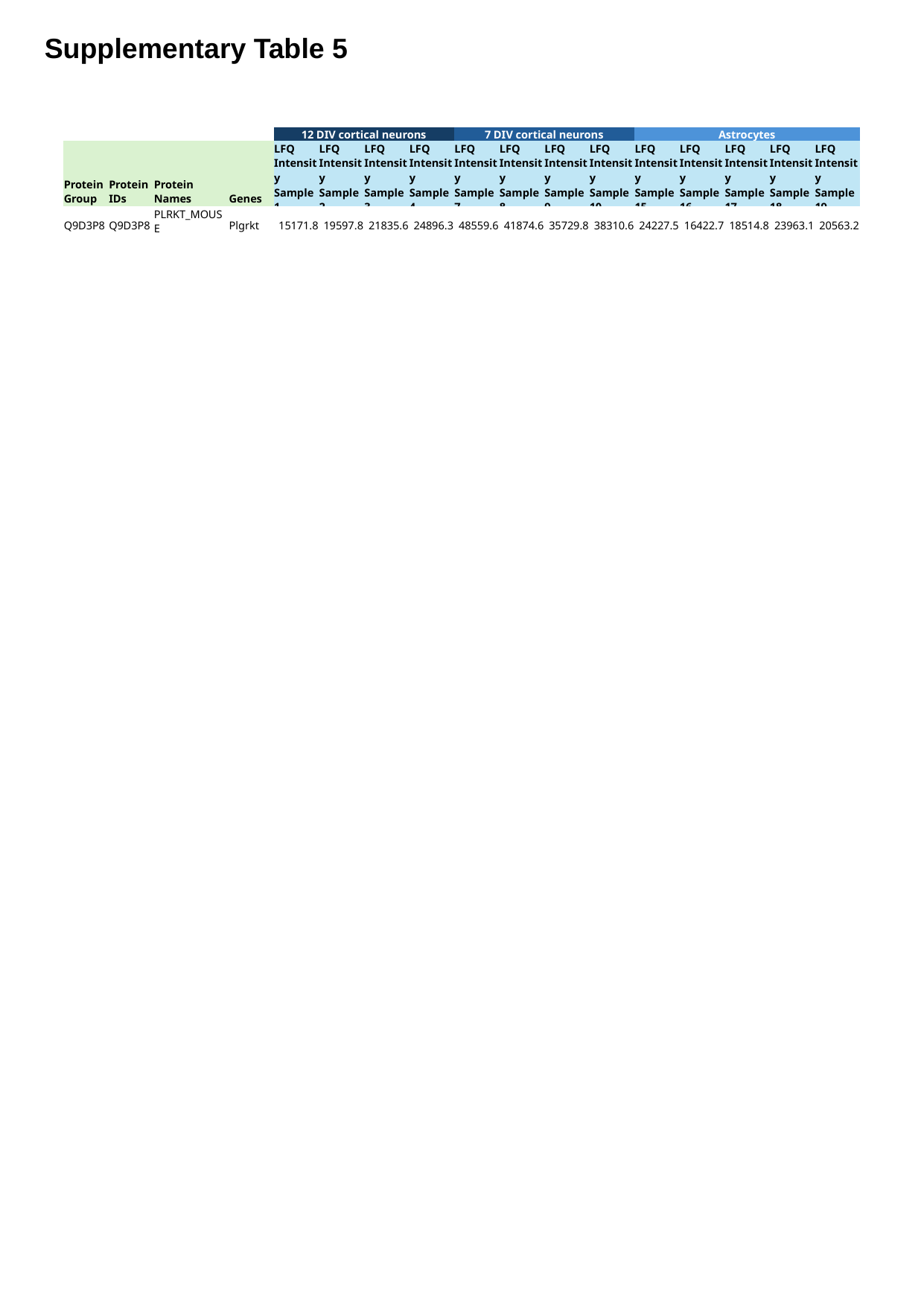

Supplementary Table 5
| | | | | 12 DIV cortical neurons | | | | 7 DIV cortical neurons | | | | Astrocytes | | | | |
| --- | --- | --- | --- | --- | --- | --- | --- | --- | --- | --- | --- | --- | --- | --- | --- | --- |
| Protein Group | Protein IDs | Protein Names | Genes | LFQ Intensity Sample 1 | LFQ Intensity Sample 2 | LFQ Intensity Sample 3 | LFQ Intensity Sample 4 | LFQ Intensity Sample 7 | LFQ Intensity Sample 8 | LFQ Intensity Sample 9 | LFQ Intensity Sample 10 | LFQ Intensity Sample 15 | LFQ Intensity Sample 16 | LFQ Intensity Sample 17 | LFQ Intensity Sample 18 | LFQ Intensity Sample 19 |
| Q9D3P8 | Q9D3P8 | PLRKT\_MOUSE | Plgrkt | 15171.8 | 19597.8 | 21835.6 | 24896.3 | 48559.6 | 41874.6 | 35729.8 | 38310.6 | 24227.5 | 16422.7 | 18514.8 | 23963.1 | 20563.2 |

## Slide 6
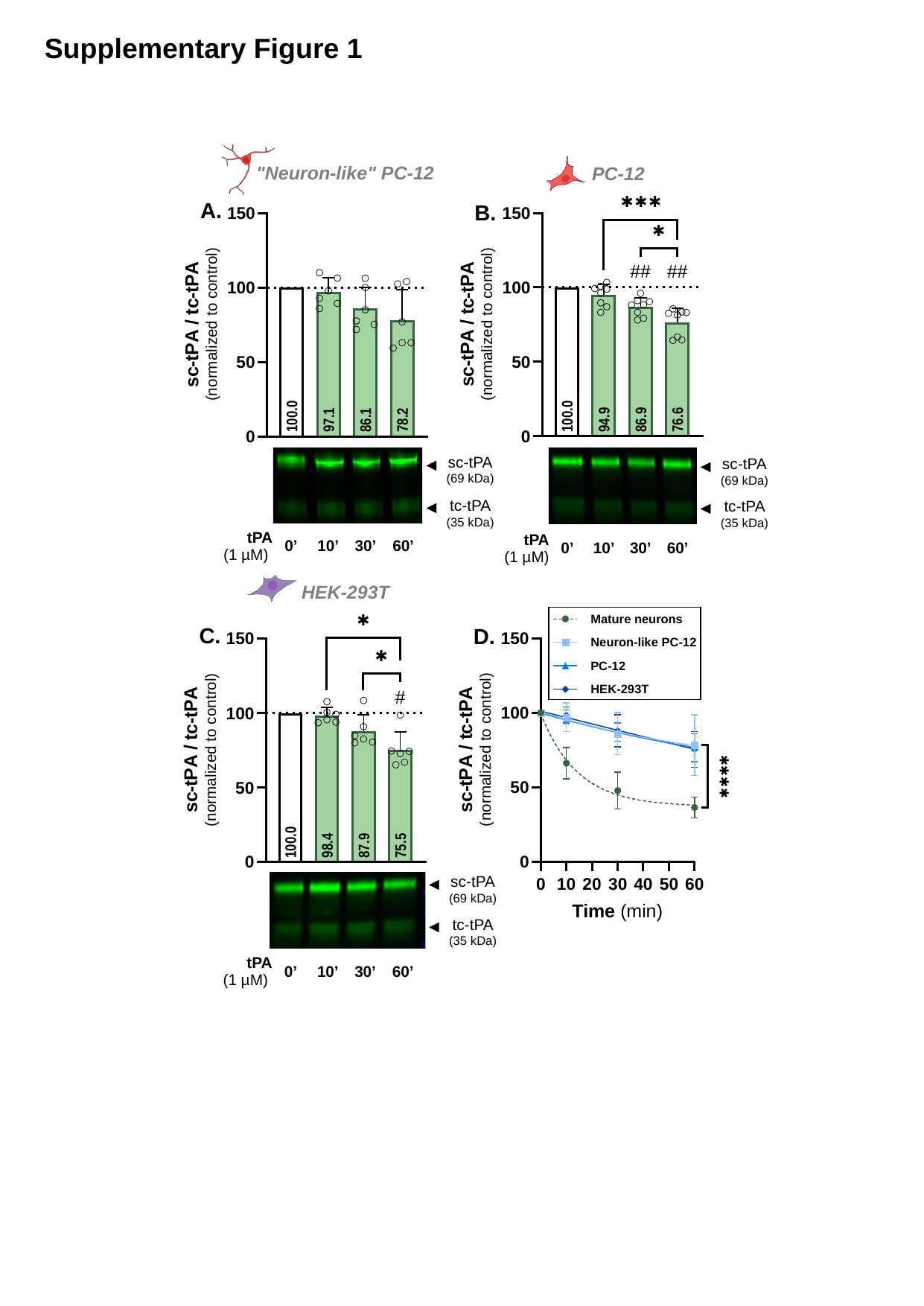

Supplementary Figure 1
"Neuron-like" PC-12
PC-12
A.
B.
sc-tPA
(69 kDa)
◄
sc-tPA
(69 kDa)
◄
◄
tc-tPA
(35 kDa)
◄
tc-tPA
(35 kDa)
| tPA (1 µM) | 0’ | 10’ | 30’ | 60’ |
| --- | --- | --- | --- | --- |
| tPA (1 µM) | 0’ | 10’ | 30’ | 60’ |
| --- | --- | --- | --- | --- |
HEK-293T
C.
D.
sc-tPA
(69 kDa)
◄
◄
tc-tPA
(35 kDa)
| tPA (1 µM) | 0’ | 10’ | 30’ | 60’ |
| --- | --- | --- | --- | --- |

## Slide 7
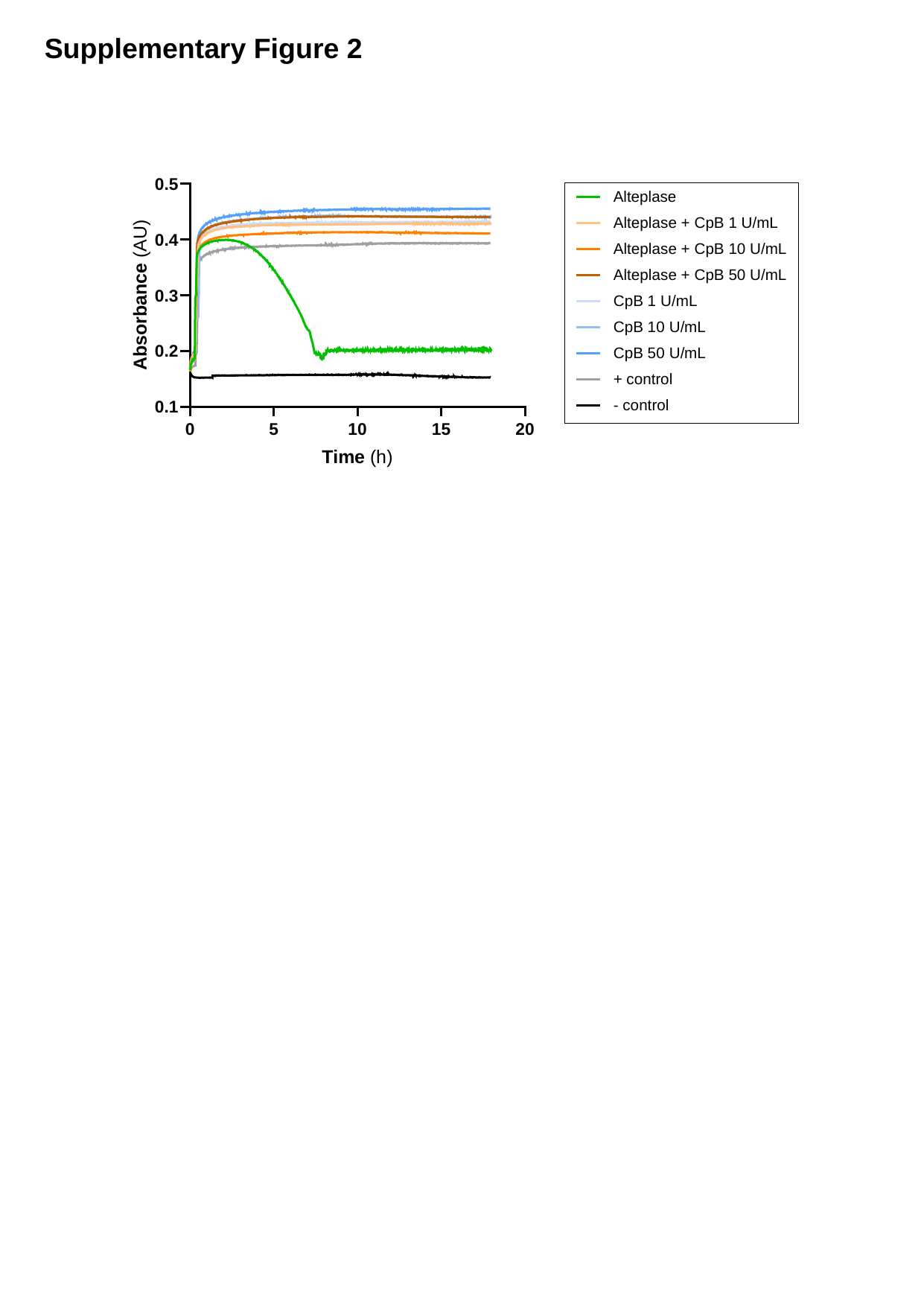

Supplementary Figure 2

## Slide 8
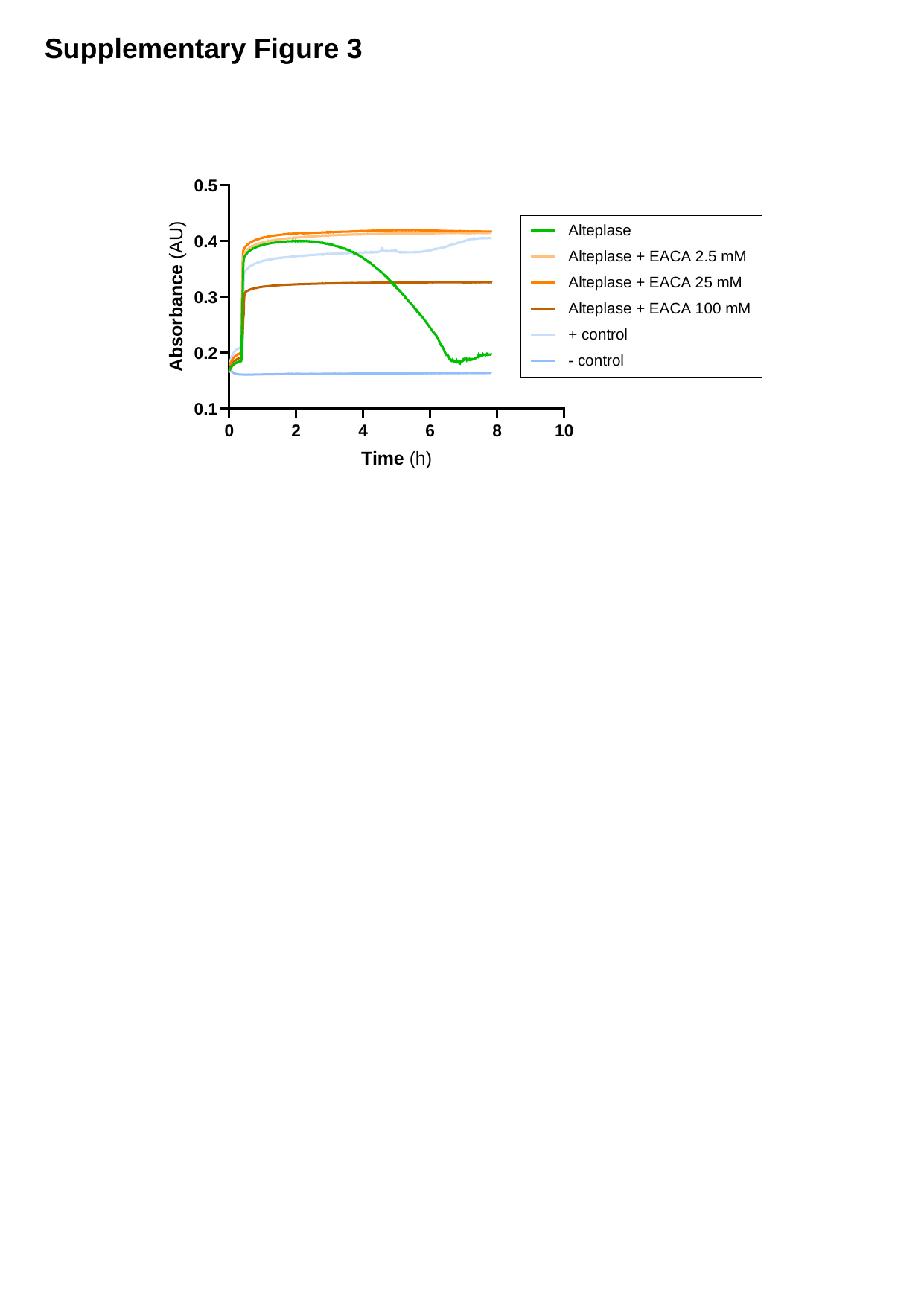

Supplementary Figure 3

## Slide 9
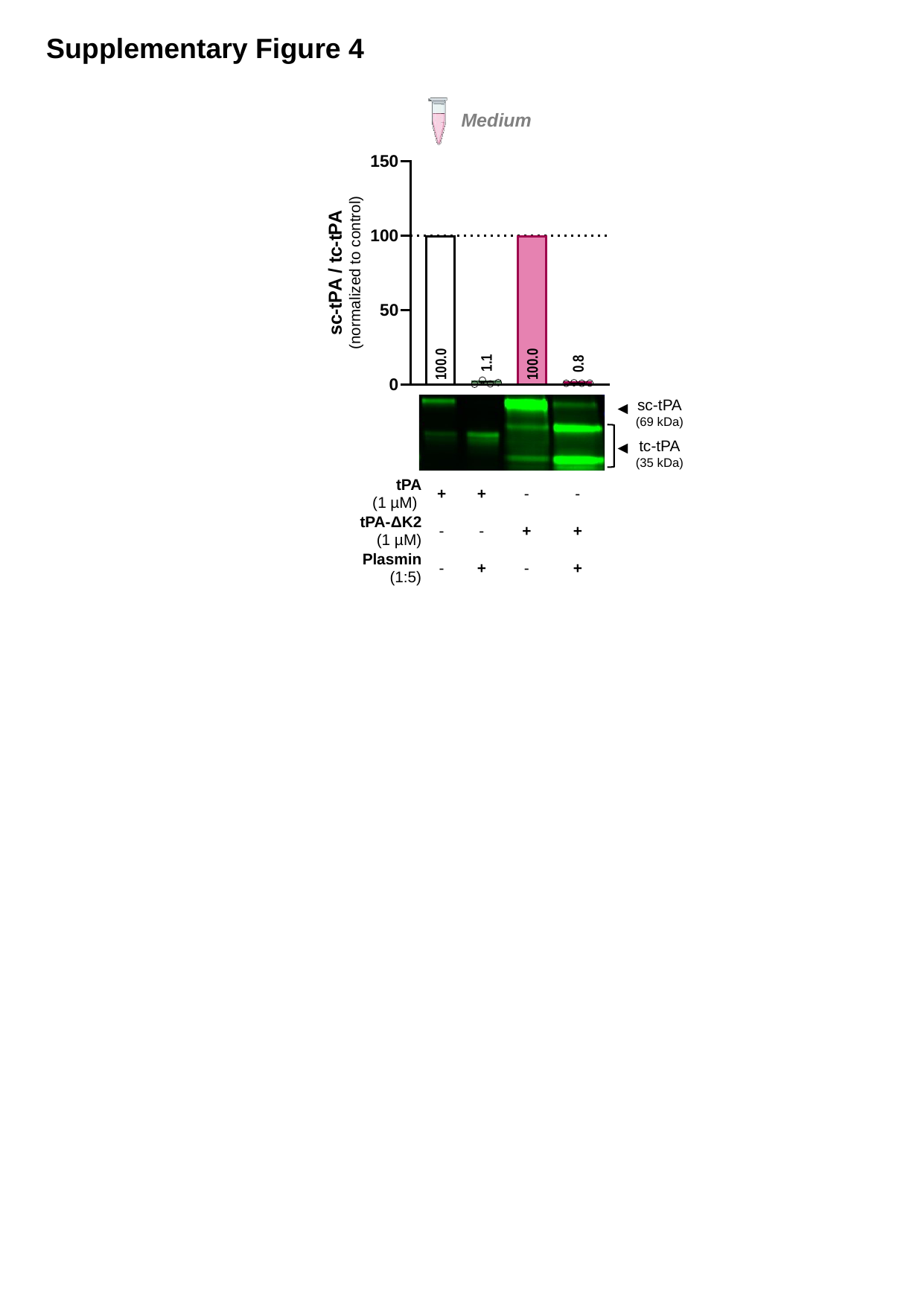

Supplementary Figure 4
Medium
sc-tPA
(69 kDa)
◄
◄
tc-tPA
(35 kDa)
| tPA (1 µM) | + | + | - | - |
| --- | --- | --- | --- | --- |
| tPA-ΔK2 (1 µM) | - | - | + | + |
| Plasmin (1:5) | - | + | - | + |
