# Supplementary material for: Neuronal conversion of single-chain tissue-type plasminogen activator into its two-chain form: implications in neurodevelopment, learning, and memory
Source: Cell Death Dis. 2025 Nov 7;16(1):811. doi: 10.1038/s41419-025-08132-8 (PMC12594754; doi:10.1038/s41419-025-08132-8)
Supplement: Supplementary file 3 — Supplemenraty legends [file 41419_2025_8132_MOESM3_ESM.docx]

**SUPPLEMENTARY LEGENDS**

**Supplementary Table 1. Culture media composition.** Summary table of media composition used for the culture of mouse primary cortical neurons (5-5-1 and neurobasal models), mouse primary astrocytes, mouse primary endothelial cells, “neuron-like” differentiated PC-12, PC-12, and HEK-293T.

**Supplementary Table 2. Cleavage kinetics statistical comparisons.** Summary table of cleavage kinetics statistical comparisons for the studied experimental conditions. Ratio values were plotted for comparison of their mean initial slopes (*** *p* < 0.001; **** *p* < 0.0001; F test).

**Supplementary Table 3. Additional targets studied in tPA cleavage.** Summary table of the different modulators tested to explore additional actors’ involvement in neuronal cleavage of tPA.

**Supplementary Table 4. Both CpB sensitive and insensitive plasminogen receptors bind to tPA after incubation on mature cortical neurons.** Mass-spectrometry based proteomic analysis of membrane and cytosolic proteins detected bound to biotinylated tPA pulled-down after incubation on mature cortical neurons for 60 minutes. Data are represented as label-free intensities (LFQ) representing the intensity of corresponding peptides; *n* = 1.

**Supplementary Table 5. Plg-RKT is expressed in both cortical neurons and non-cleaving cells.** Mass-spectrometry based proteomic analysis of membrane proteins detected in primary mature cortical neurons (12 DIV), immature cortical neurons (7 DIV), and primary astrocytes. Data are represented as label-free intensities (LFQ representing the intensity of corresponding peptides *n* = 4 (12 and 7 DIV), *n* = 5 (astrocytes).

**Supplementary Figure 1.** **Cleavage of sc-tPA into tc-tPA is specific to cortical neurons. A.** Densitometric quantification of the ratio sc-tPA^488^/tc-tPA^488^ (1 µM) normalized to stain free in % of control condition in “neuron-like” differentiated PC-12 and representative electrophoresis. **B.** Densitometric quantification of the ratio sc-tPA^488^/tc-tPA^488^ (1 µM) normalized to stain free in % of control in PC-12 and representative electrophoresis. **C.** Densitometric quantification of the ratio sc-tPA^488^/tc-tPA^488^ (1 µM) normalized to stain free in % of control condition in HEK-293T and representative electrophoresis. **D.** Kinetic representation of the ratio sc-tPA^488^/tc-tPA^488^ in “neuron-like” differentiated PC-12, PC-12, and HEK-293T cells compared with the previously demonstrated mature cortical neurons. Data are represented as mean ± SD; *n* = 6 (A-D); * *p* < 0.05; *** *p* < 0.001; **** *p* < 0.0001; # *p* < 0.05 compared to control; ## *p* < 0.01 compared to control; One-sample Wilcoxon test, Mann-Whitney test, F test.

**Supplementary Figure 2. CpB inhibits tPA thrombolytic activity.** *In vitro* clot lysis time assays in human platelet-poor plasma (PPP). Clotting was initiated with calcium chloride, in the presence of rtPA (Alteplase) and/or CpB. Positive control consists in PPP + calcium chloride, negative control consists in PPP only. Data are represented as mean curves; 6 pooled PPP; *n* = 3.

**Supplementary Figure 3. EACA inhibits tPA thrombolytic activity.** *In vitro* clot lysis time assays in human platelet-poor plasma (PPP). Clotting was initiated with calcium chloride, in the presence of rtPA (Alteplase) and/or EACA. Positive control consists in PPP + calcium chloride, negative control consists in PPP only. Data are represented as mean curves; 6 pooled PPP; *n* = 3.

**Supplementary Figure 4.** **The mutant tPA-*Δ*K2^488^ is cleavable by plasmin.** Densitometric quantification of the ratio sc-tPA-*Δ*K2^488^/tc-tPA-*Δ*K2^488^ (1 µM) normalized to stain free in % of control condition in the presence of plasmin and representative electrophoresis.
